# Mur ligase F as a new target for the flavonoids quercitrin, myricetin, and (–)-epicatechin

**DOI:** 10.1007/s10822-023-00535-z

**Published:** 2023-10-05

**Authors:** Martina Hrast Rambaher, Irena Zdovc, Nina Kočevar Glavač, Stanislav Gobec, Rok Frlan

**Affiliations:** 1 Department of Pharmaceutical Chemistry, Faculty of Pharmacy, University of Ljubljana, Aškerčeva 7, 1000 Ljubljana, Slovenia; 2 Veterinary Faculty, Institute of Microbiology and Parasitology, University of Ljubljana, Gerbičeva ul. 60, Ljubljana, Slovenia; 3 Department of Pharmaceutical Biology, Faculty of Pharmacy, University of Ljubljana, Aškerčeva 7, 1000 Ljubljana, Slovenia

**Keywords:** MurF, Quercitrin, Myricetin, (–)-epicatechin, Antibacterial

## Abstract

MurC, D, E, and F are ATP-dependent ligases involved in the stepwise assembly of the tetrapeptide stem of forming peptidoglycan. As highly conserved targets found exclusively in bacterial cells, they are of significant interest for antibacterial drug discovery. In this study, we employed a computer-aided molecular design approach to identify potential inhibitors of MurF. A biochemical inhibition assay was conducted, screening twenty-four flavonoids and related compounds against MurC-F, resulting in the identification of quercitrin, myricetin, and (–)-epicatechin as MurF inhibitors with IC_50_ values of 143 µM, 139 µM, and 92 µM, respectively. Notably, (–)-epicatechin demonstrated mixed type inhibition with ATP and uncompetitive inhibition with d-Ala-d-Ala dipeptide and UM3DAP substrates. Furthermore, *in silico* analysis using Sitemap and subsequent docking analysis using Glide revealed two plausible binding sites for (–)-epicatechin. The study also investigated the crucial structural features required for activity, with a particular focus on the substitution pattern and hydroxyl group positions, which were found to be important for the activity. The study highlights the significance of computational approaches in targeting essential enzymes involved in bacterial peptidoglycan synthesis.

**Graphical abstract:**

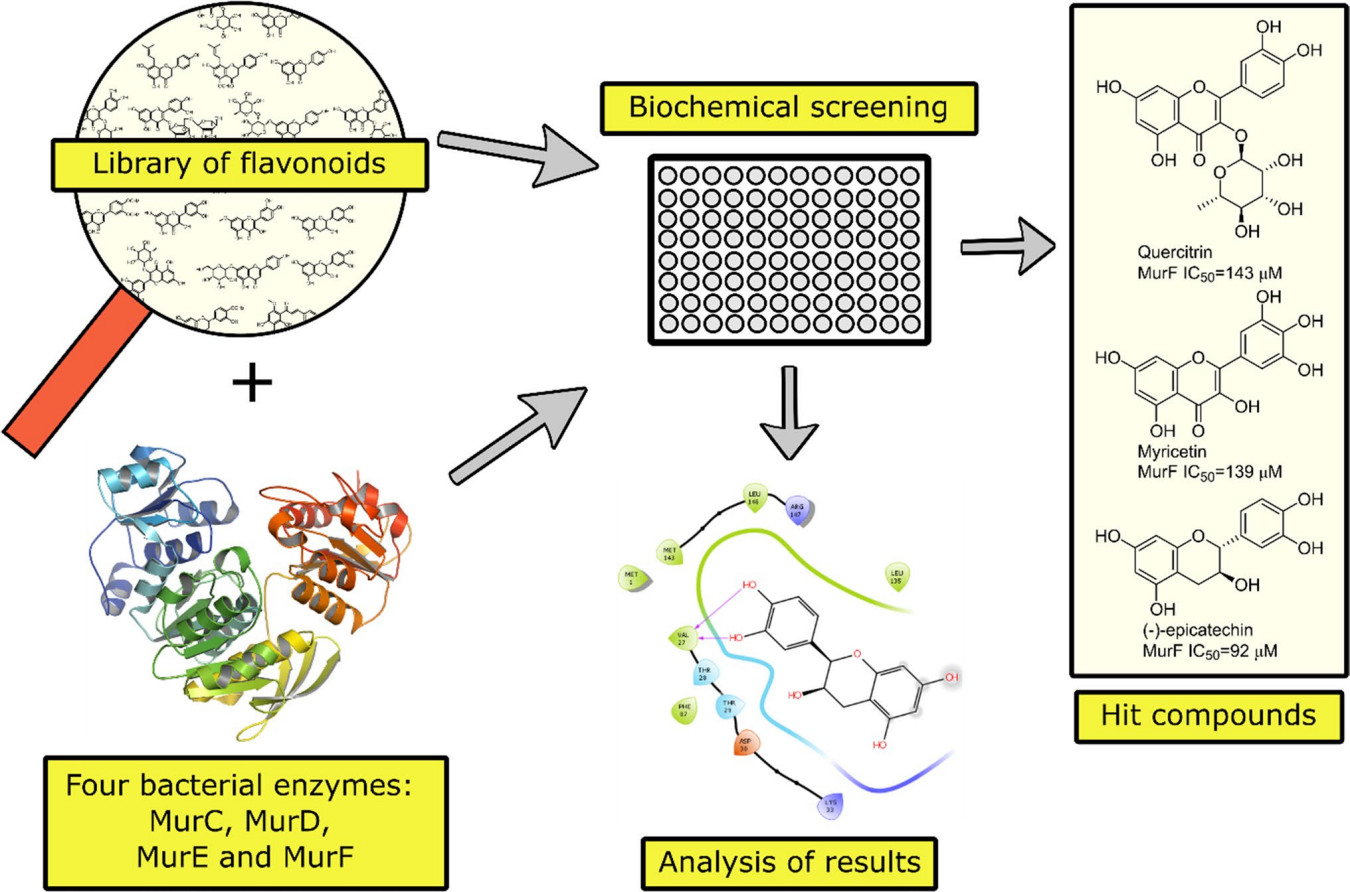

**Supplementary Information:**

The online version contains supplementary material available at 10.1007/s10822-023-00535-z.

## Introduction

Enzymes involved in peptidoglycan (PG) synthesis have been a cornerstone of modern antibiotic therapy for over 80 years [1]. Most antibiotics that inhibit peptidoglycan biosynthesis, such as β-lactams, target enzymes responsible for the extracellular steps of PG synthesis. On the other hand, the enzymes involved in the cytoplasmic steps remain largely underexplored pharmacologically as antibacterial targets. Currently, only two intracellular enzymes, UDP-N-acetylglucosamine-enolpyruvyl transferase (MurA) and d-alanine: d-alanine ligase (Ddl), have been clinically validated as antibacterial targets by fosfomycin [2] and d-cycloserine [3], respectively.

The stepwise intracellular assembly of the tetrapeptide stem in forming peptidoglycan is catalyzed by a series of four ATP-dependent enzymes known as Mur ligases (MurC, D, E, and F). This assembly involves the addition of l-alanine (MurC), d-glutamic acid (MurD), a diamino acid, usually meso-diaminopimelic acid or l-lysine (MurE), and the dipeptide d-Ala-d-Ala (MurF) to the d-lactoyl group of UDP-MurNAc. All four enzymes have great potential as antibacterial targets because they are highly conserved, essential for growth and virulence, and are only found in bacterial cells [4]. Although several inhibitors targeting each of these enzymes have been reported in the last two decades, they have generally exhibited only weak antibacterial activity, likely due to their low cell wall penetration (For a comprehensive review, see Hrast et al. [5]).

For more than a century, one successful strategy for sourcing novel lead compounds has been the use of polyphenolic phytochemicals, particularly flavonoids. These compounds are well-known for the color they impart to fruits and vegetables, as well as for their health benefits, including anti-inflammatory, antidiabetic, anti-cancer, and immunomodulatory effects. They also act as antioxidants, such as metal chelators and free radical scavengers, and provide protection against the damaging effects of reactive oxygen species. Given these properties, many flavonoids have potential benefits in the prevention of chronic diseases such as osteoporosis, atherosclerosis, neurodegenerative diseases, and cancers caused by free radical damage [6].

Out of the 9000 flavonoids identified so far, many exhibit noteworthy antibacterial activity against a broad range of common Gram-positive and Gram-negative pathogens in vitro. While these effects are likely due to a complex mechanism that acts on multiple bacterial targets, comprehending how flavonoids interact with these targets could provide insight into their potential mechanism of action and the necessary structural requirements for inhibition [7].

A large group of bacterial and mammalian enzymes can be inhibited by flavonoids, with many of them being ATP-binding enzymes [8]. In most cases, the action of flavonoids on these enzymes has been shown to be competitive with ATP, although other binding modes such as noncompetitive mode have also been reported, e.g., for Src family kinases [9]. Although flavonoids have a promiscuous nature, limiting their potential use as drugs per se, some degree of specificity has been observed even for kinases [10]. Nevertheless, flavonoids are still considered useful templates for developing more potent and selective analogs [11]. Furthermore, it is acknowledged that valuable pharmacophore information can be obtained from structure-activity analysis and the study of interactions between flavonoids and ATP-binding sites. This information can then be utilized to construct a pharmacophore model suitable for screening larger chemical libraries [12].

**Fig. 1:**
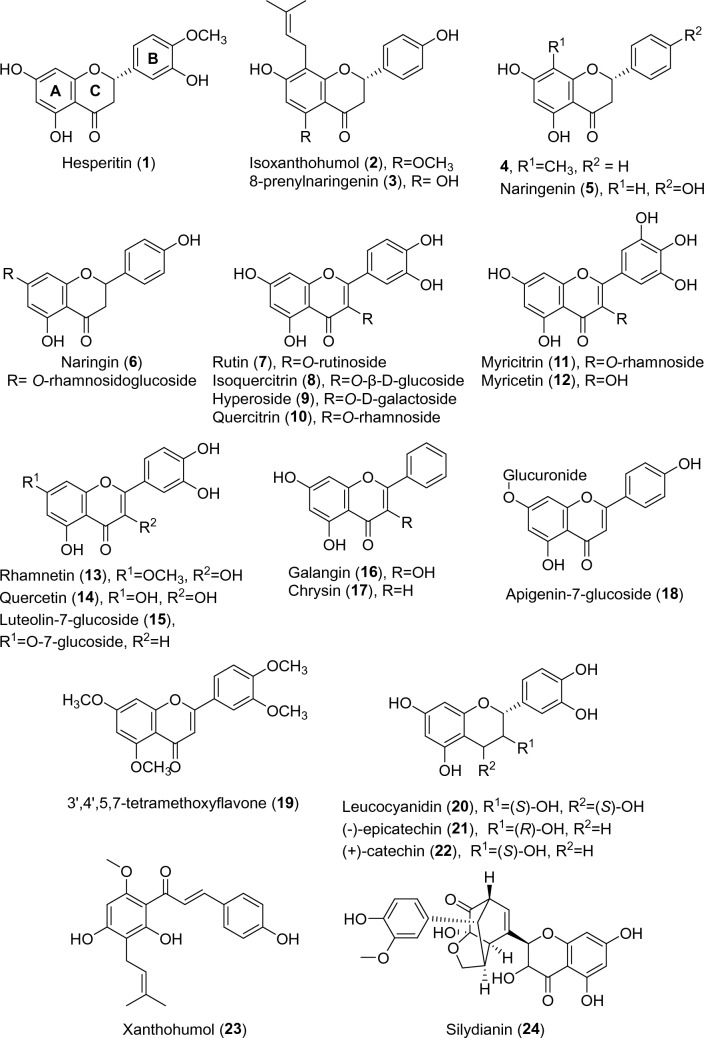
Structures of selected flavonoids and related compounds. Nomenclature of flavonoid ring system is also shown. Created with ChemDraw 19.0

A previous study by Wu et al. reported that quercetin and apigenin inhibit d-Ala:d-Ala ligase, an ATP-dependent enzyme involved in the intracellular steps of PG synthesis [13]. Building upon this study, our research focused on the four ATP-dependent enzymes involved in the intracellular steps of PG synthesis, namely Mur ligases C, D, E, and F. The main objective of this study was to investigate whether the antibacterial properties of selected polyphenols are related to the inhibition of these ligases, to discover new inhibitors of these enzymes with flavonoid structure, and to provide additional information on the structural properties required for the development of more effective inhibitors. We screened a group of 24 flavonoids and related compounds, including silymarin, a *Silybum marianum* L. (milk thistle) extract with flavolignans as the main components, silydianin (**24**), a flavolignan of the silymarin extract, and xanthohumol (**23**), a chalconoid (Fig. 1), for their ability to inhibit Mur ligases C, D, E and F. Our results showed that Mur ligases C, D, and E were largely insensitive to all 25 samples, whereas MurF was inhibited by three compounds. It was also demonstrated that although several compounds inhibited the growth of *S. aureus*, their activity was not due to the inhibition of the assayed Mur ligases.

## Materials and methods

### Chemicals

The polyphenols were provided by the The Department of Pharmaceutical Biology, Faculty of Pharmacy, University of Ljubljana, and originally sourced from various companies including Alfa–Aesar (**19**), APIN Chemicals Ltd. (**2**), Carl Roth (**7**, **9**, **15**, **16**, **18**), ChromaDex (**23**), Fluka (**4**, **6**, **10**, **11**, **12**, **20**), Janssen Chimica (**21**), Serva (**5**, **17**, **22**), Sigma–Aldrich (**1**, **8**, **13**, **24**), LGC Standards (Quercetin) and Nookhandeh Institut für Naturstoffchemie Homburg (**3**). Silymarin was extracted from the Silliver® tablets and all compounds were stored in the dark in a refrigerator to protect them from light and possible decomposition. Prior to the biological assay, all compounds were dissolved in dimethyl sulfoxide (DMSO and stored at − 20 °C. All other chemicals were of ultra-pure analytical grade and were purchased from Sigma–Aldrich or Acros Chemical Company.

### Computational analysis

#### Protein preparation

In *E. coli*, only the apo form of MurF crystal structures are available (PDB Access Code 1GG4). Therefore, a homology model of the enzyme was constructed using SWISS-MODEL [14], a fully automated protein structure homology-modelling server. The protein sequence of MurF from *E. coli* was obtained from the UniProtKB database [15] with the code P11880 and an automated search was conducted to identify the most similar templates. The resulting templates were subsequently prioritized according to the quality of the resultant models. Among these, the crystal structure of MurF from *A. baumanii* (PDB Access Code 4QF5) which contained bound ATP and was the highest-ranked crystal structure, was chosen as a template based on chain A.

Although the two structures share only 40.4% identity, the ATP-binding pocket is highly conserved, and 15 of the 18 residues are identical. The initial structure of the ternary complex was further prepared using the Protein Preparation Wizard in Maestro, which is described below. Finally, the accuracy of the model was validated using three online tools: ERRAT [16], PROSA [17], and RAMPAGE [18].

#### Protein preparation wizard

A protein structure was generated using the SWISS-MODEL [14] and prepared using Schrödinger’s Protein Preparation Wizard, which is implemented in the Schrödinger Suite [19]. Water and other co-crystallized molecules, except for magnesium ions, were removed. Bond orders were automatically assigned, and hydrogen atoms were added. Selenomethionines were converted to methionines, and missing side chains were added. Water beyond the 3 Å radius of heteroatoms and with fewer than 3 hydrogen bonds to non-waters was removed. Heteroatoms were protonated at pH 7.0. Finally, the impred script was used to perform a constrained minimization of the protein with a maximum root mean square deviation (RMSD) of 0.30 Å.

#### SiteMap analysis

SiteMap is a software tool used to identify and evaluate binding sites. It is implemented in Schrödinger’s Maestro software [19]. We chose SiteMap because it has been validated using a set of 538 crystal structures [20]. SiteMap uses interaction energies between the protein and grid probes to identify energetically favorable sites. The program generates a grid of points in a three-step process, and then scores the sites based on their energetic properties. Up to 10 potential binding sites were allowed, and only those containing at least 15 points per site were considered. A more restrictive definition of hydrophobicity was used, along with a standard grid. Site maps located 4 Å or more from the nearest site points were truncated. The developers proposed a SiteScore value of 0.80 as a cutoff value to distinguish between sites that can and cannot bind ligands. We identified a total of seven binding sites, and selected the first binding sites with a SiteScore value greater than 0.8 for further analysis and grid box preparation.

#### Preparation of the grid box

The SiteMap software was used to identify binding sites, which were then used as a reference to define two boxes enclosing the three identified binding sites: (a) site 1, which is an ATP binding site with xyz coordinates of 7 Å, − 5 Å, 17 Å and a diameter of 27 Å, and (b) sites 2 and 3. Site 2 is an UDP-MurNAc-tripeptide (UM3DAP) binding site. A grid box enclosing both binding sites was defined with xyz coordinates of 6 Å, 2 Å, − 11 Å and a diameter of 24 Å. No constraints were applied during the process.

#### Preparation of ligands

Molecules were drawn using ChemDraw18 (PerkinElmer, MA, USA), and OpenBabel [21] was used to covert the structures to SMILES format. These structures were then imported into the Maestro program [19] and further prepared using LigPrep [22] from the Schrödinger Suite. The molecular conformations were generated using the OPLS4 force field, and the protonation states were adjusted using Epik [23] at a target pH of 7 ± 2. The resulting conformers were then stored in Maestro format for subsequent docking. The conformers were finally stored in Maestro format for further docking.

#### Docking procedure

Glide software [24] from Schrödinger Suite was used for docking in standard precision mode (SP). Active flavonoids were docked using the two previously defined lattice boxes. To enhance the flexibility of the nonpolar regions within the ligand, we applied a scaling factor of 0.8 to the van der Waals radii of ligand atoms. Additionally, ligand atoms possessing partial atomic charges with an absolute value below the specified cutoff of 0.15 were subjected to this scaling process. This approach allows for a controlled reduction in the nonpolar volume of the ligand while maintaining interactions with other atoms intact. Flexible ligand sampling was used and ring conformations were sampled. Epik state penalties were added to the docking score, intramolecular hydrogen bonding was rewarded and conjugated π groups had enhanced planarity. No constraints were used and post-docking energy minimization was performed with strain correction terms.

#### Visualization of results

All analyses and visualizations of the docking poses were performed using Schrodinger’s Maestro. [19] The original ATP-binding coordinates were used to compare ATP and flavonoid binding positions. To visualize the UM3DAP binding region another PDB structure (PDB Access Code 4QDI) with bound UDP was aligned to our model and then UDP was extracted for comparison with flavonoid binding position.

### Biochemical analysis

#### MurC-F inhibition assay

Inhibition of the Mur ligases was determined using the Malachite green assay, with slight modifications [25]. The mixtures for the respective Mur ligase assays had a final volume of 50 µL, which contained 100 µM of each tested compound dissolved in DMSO, added to: (1) MurC: 50 mM Hepes, pH 8.0, 5 mM MgCl_2_, 0.005% Triton X-114, 120 µM l-Ala, 120 µM UDP-*N*-acetylmuramic acid, 450 µM ATP, and 50 nM purified *E. coli* MurC [26]; (2) MurD: 50 mM Hepes, pH 8.0, 5 mM MgCl_2_, 0.005% Triton X-114, 100 µM d-Glu, 80 µM UDP-*N*-acetylmuramoyl-l-alanine (UMA), 400 µM ATP, and 150 nM purified *E. coli* MurD [27]; (3) MurE: 50 mM Hepes, pH 8.0, 15 mM MgCl_2_, 0.005% Triton X-114, 60 µM *meso*-diaminopimelic acid, 100 µM UDP-*N*-acetylmuramoyl-l-alanine-d-glutamate, 1000 µM ATP, and 20 nM purified *E. coli* MurE [28]; (4) MurF: 50 mM Hepes, pH 8.0, 50 mM MgCl_2_, 0.005% Triton X-114, 600 µM d-Ala- d-Ala, 100 µM UDP-*N*-acetylmuramoyl-l-alanine-d-glutamate-2,6-diaminopimelic acid (UM3DAP), 500 µM ATP, and 10 nM purified *E. coli* MurF [25, 29]. In all cases, the final concentration of DMSO was 5% (v/v).

After incubation for 15 min at 37 °C, the enzyme reaction was terminated by the addition of 100 µM Biomol green reagent®, and the absorbance was measured at 650 nm after 5 min. Experiments to determine the RA run in triplicate. Residual activities were calculated with respect to control assays without the tested compounds, but with the 5% DMSO carrier. The IC_50_ values were determined by measuring the residual activities at seven different compound concentrations, and they represent the concentration of the compound at which the residual activity was 50%. IC_50_ values were obtained by plotting the residual enzyme activities against the applied inhibitor concentrations, fitting the experimental data to the 4-parameter Hill equation: $Y = {\text{Bottom}} - \frac{{{\text{Top}} - {\text{Bottom}}}}{{1 + 10^{{\left( {\log \left( {{\text{IC}}_{{50}}  - {\text{X}}} \right) \times {\text{Hill}}\,{\text{Slope}}} \right)}} }}$, where X is the logarithm of the inhibitor concentration, and Y is the residual activity. The IC_50_ values were determined in three independent experiments. GraphPad Prism 8.2.0 (GraphPad Software, San Diego, CA, USA) was used for the fitting procedure.

#### Assay interference screen

To assess the potential interference of compounds using the malachite green-based assay, the following procedure was conducted:

Compounds, dissolved in DMSO, at 100 µM (5% DMSO, v/v), were combined with a substrate mixture (50 µL; containing 50 mM Hepes pH 8.0, 5 mM MgCl_2_, and 500 µM ATP) along with Biomol® reagent (100 µL). After incubating the mixture for 5 min at room temperature, the absorbance was measured at 650 nm. All experiments were performed in triplicate. A blank sample was prepared under identical conditions, utilizing only DMSO (5%, v/v). Compounds exhibiting a difference in absorbance greater than 0.1 (A_cpd_ − A_blank_ ≥ 0.1) were categorized as causing interference with the assay.

#### Steady-state kinetic analysis of compound **21**

For compound **21**, Ki values were determined against MurF from *E. coli.* Ki determinations were performed under similar conditions to those described for the inhibition assay, where the different concentrations of one substrate and a fixed concentration of the other two were used. First, the concentration of UM3DAP was varied (25, 50, 100, 200 µM) at fixed ATP (500 µM) and d-Ala-d-Ala (600 µM)), then the concentration of d-Ala-d-Ala was varied (50, 100, 200, 400, 600 µM) at fixed ATP (500 µM) and UM3DAP (100 µM), and finally, the concentration of ATP was varied (50, 100, 350, 500 µM) at fixed UM3DAP (100 µM) and d-Ala-d-Ala (600 µM). The concentrations of 21 were 0, 25, 50, 75, 100, 200, 350 and 500 µM and the concentration of the MurF was 20 nM. After a 15 min incubation, 100 µM Biomol green reagent® was added, and the absorbance was read at 650 nm after 5 min. All experiments were run in triplicate.

The data were analysed using the SigmaPlot 12.0 software. The initial velocities were fitted to competitive, non-competitive, uncompetitive and mixed enzyme inhibition. The Ki and mode of inhibition from the best ranking model were used, as provided by the software.

#### Antibacterial assays

Antimicrobial testing was performed by the broth microdilution method in 96-well plates following the Clinical and Laboratory Standards Institute guidelines and European Committee on Antimicrobial Susceptibility Testing recommendations (Clinical and Laboratory Standards Institute). Bacterial suspensions equivalent to 0.5 McFarland turbidity standard were diluted with cation-adjusted Mueller–Hinton broth with TES buffering (ThermoFisher Scientific), for a final inoculum of 10^5^ CFU/ mL. Compounds dissolved in DMSO and the inoculum were mixed and incubated for 20 h at 35 °C. After this incubation, the minimal inhibitory concentrations (MICs) were determined by visual inspection, as the lowest dilution of the compounds that showed no turbidity. The MICs were determined against two reference bacterial strains, *S. aureus* (ATCC 29,213) and *E. coli* (ATCC 25,922). Tetracycline was used as the positive control on every assay plate, with MICs of 0.5 and 1 µg/mL for *S. aureus* and *E. coli*, respectively. All MICs were determined in three independent experiments.

## Results

### MurC-F inhibition assay

Figure 1 displays the structures of 24 isolated flavonoids and related compounds (**1**–**24**), which were evaluated, along with a silymarin extract, for their ability to inhibit Mur ligases C, D, E, and F from *E. coli* using the Malachite green assay. The assay detects the phosphate formed in each enzyme reaction [25]. To avoid any possibility of the compounds inhibiting the enzymes due to aggregation, a detergent (0.005% Triton-X114) was added to the test system.

The results are summarized in Table 1 as residual activities at 100 µM concentration (RA) or as IC_50_ values (µM). The latter was determined only for the selected compounds that showed RA < 50% and did not interact with the assay. While RA values were solely measured at a singular concentration of 100 µM, the determination of IC_50_ values involved assessing residual enzyme activities across seven distinct concentrations. These activities were then plotted against their corresponding inhibitor concentrations. Subsequently, the empirical data underwent a fitting procedure utilizing the robust 4-parameter Hill equation. As a consequence of this process, it is not uncommon for the IC_50_ values of certain compounds to exceed 100 µM.

**Table 1:** Results of in vitro inhibition of **1**–**25** on selected Mur ligases C, D, E and F together with minimal inhibitory concentration (MIC, mM) against *E. coli* and *S. aureus*

No.	RA (%) @ 100 µM or IC_50_				MIC (mM)	
	MurC (%)	MurD (%)	MurE	MurF	*E.coli*	*S.aureus*
1	100 ± 9	94 ± 9	100 ± 10%	87 ± 8%	> 0.25	> 0.25
2	86 ± 8	100 ± 10	77 ± 7%	78 ± 9%	> 0.25	**= 0.25**
3	95 ± 10^b^	100 ± 8	87 ± 9%	71 ± 7%	> 0.25	**= 0.062**
4	91 ± 9	97 ± 8	100 ± 9%	88 ± 9%	> 0.25	> 0.25
5	97 ± 8	100 ± 9	98 ± 9%	87 ± 9%	> 0.25	> 0.25
6	89 ± 9	95 ± 10	100 ± 9%	90 ± 8%	> 0.25	> 0.25
7	100 ± 10	97 ± 9	100 ± 10%	89 ± 10%	> 0.25	> 0.25
8	95 ± 9	100 ± 9	97 ± 8%	88 ± 9%	> 0.25	> 0.25
9	89 ± 8	100 ± 8	100 ± 8%	90 ± 8%	> 0.25	> 0.25
10	98 ± 10	77 ± 9	98 ± 9%	143 ± 18 µM Hill = 0.8	> 0.25	> 0.25
11	97 ± 7	89 ± 7	100 ± 10%	86 ± 9%	> 0.25	> 0.25
12	87 ± 8	87 ± 9	66 ± 7%	139 ± 13 µM Hill = 0.8	> 0.25	> 0.25
13	66 ± 7^a^	71 ± 8^b^	82 ± 8%	51 ± 6%	> 0.25	> 0.25
14	41 ± 5^a^	43 ± 6^a^	28 ± 4%^a^	31 ± 9 µM^a.b^ Hill = 1.0	> 0.25	**= 0.25**
15	100 ± 9	75 ± 6	100%	92 ± 9%	> 0.25	> 0.25
16	100 ± 8^a.b^	91 ± 7	100 ± 10%^a.b^	100 ± 10%	> 0.25	**= 0.25**
17	100 ± 10	71 ± 9	83 ± 8%	100 ± 8%	> 0.25	> 0.25
18	100 ± 10	90 ± 8	100 ± 9%	91 ± 8%	> 0.25	> 0.25
19	100 ± 9	100 ± 10	100 ± 9%	100 ± 9%	> 0.25	> 0.25
20	95 ± 8	91 ± 9	164 ± 14 µM^c^ Hill = 0.7	28 ± 8 µM^c^ Hill = 1.8	> 0.25	> 0.25
21	92 ± 8	94 ± 9	79 ± 8%	92 ± 13 µM Hill = 1.0	> 0.25	> 0.25
22	100 ± 10	93 ± 8	88 ± 8%	100 ± 10%	> 0.25	> 0.25
23	86 ± 8^a^	91 ± 10	82 ± 7%	72 ± 8%	> 0.25	**= 0.031**
24	100 ± 7	95 ± 9	93 ± 9%	99 ± 7%	> 0.25	> 0.25
25^d^	89 ± 9	99 ± 8	86 ± 9%	53 ± 6%	> 0.25	> 0.25

In vitro inhibitory values may not necessarily correlate with the experimental MIC against selected bacterial strains due to possible off-targeting effects

The data represent the mean ± standard deviation of three independent experiments

^a^Interaction with test

^b^High background

^c^Poor solubility

^d^Silymarin extract

Most flavonoids tested at concentrations up to 100 µM did not inhibit Mur ligases C, D, and E. Quercetin (**14**) displayed activity against all four ligases but interacted with the assay and had a high background, so it could be considered a false positive. Leucocyanidin (**20**) inhibited both MurE and MurF with IC_50_ values of 164 µM and 28 µM, respectively. However, this result was also deemed unreliable due to the compound’s poor solubility under the assay conditions, which could have affected the assay result. In addition, compounds such as **9**, **15**, **19**, **22** and **24** increased the absorbance at 650 nm at 100 µM, leading to less reliable determination of RAs. In contrast, quercitrin (**10**), myricetin (**12**) and (–)-epicatechin (**21**) inhibited MurF with IC_50_ values of 143 µM, 139 µM and 92 µM, respectively.

### Antibacterial assays

All flavonoids were tested for their ability to inhibit the growth of *E. coli* and *S. aureus* using the broth microdilution method, and the results are shown in Table 1. Among all the flavonoids tested, only compounds **2, 3**, **14**, **16** and **23** were found to inhibit the growth the growth of *S. aureus.* Xanthohumol (**23**) exhibited the highest inhibitory potential with a MIC of 31 µM. The remaining flavonoids did not show any significant inhibition of bacterial growth under the assay conditions.

### Binding kinetics

To further investigate the mechanism by which active flavonoids inhibit MurF, inhibition kinetics were performed with (–)-epicatechin (**21**) in relation to UM3DAP, d-Ala-d-Ala and ATP. (–)-epicatechin (**21**) was found to be uncompetitive for UM3DAP and the d-Ala-d-Ala dipeptide with Ki values of 70.1 and 58.2 µM, respectively. A mixed inhibition was observed for ATP, with a Ki of 28.5 µM; the results are summarized in Table 2.

**Table 2:** Binding kinetics

Substrate	Mechanism	Ki (µM)	R^2^
UM3DAP	Uncompetitive (partial)	70.1 ± 4.0 µM	0.97887
d-Ala-d-Ala	Uncompetitive (partial)	58.2 ± 5.1 µM	0.94735
ATP	Mixed (partial)	28.5 ± 2.7 µM α = 1.5 β = 0.4545	0.98264

### Analysis of binding sites with SiteMap

**Fig. 2:**
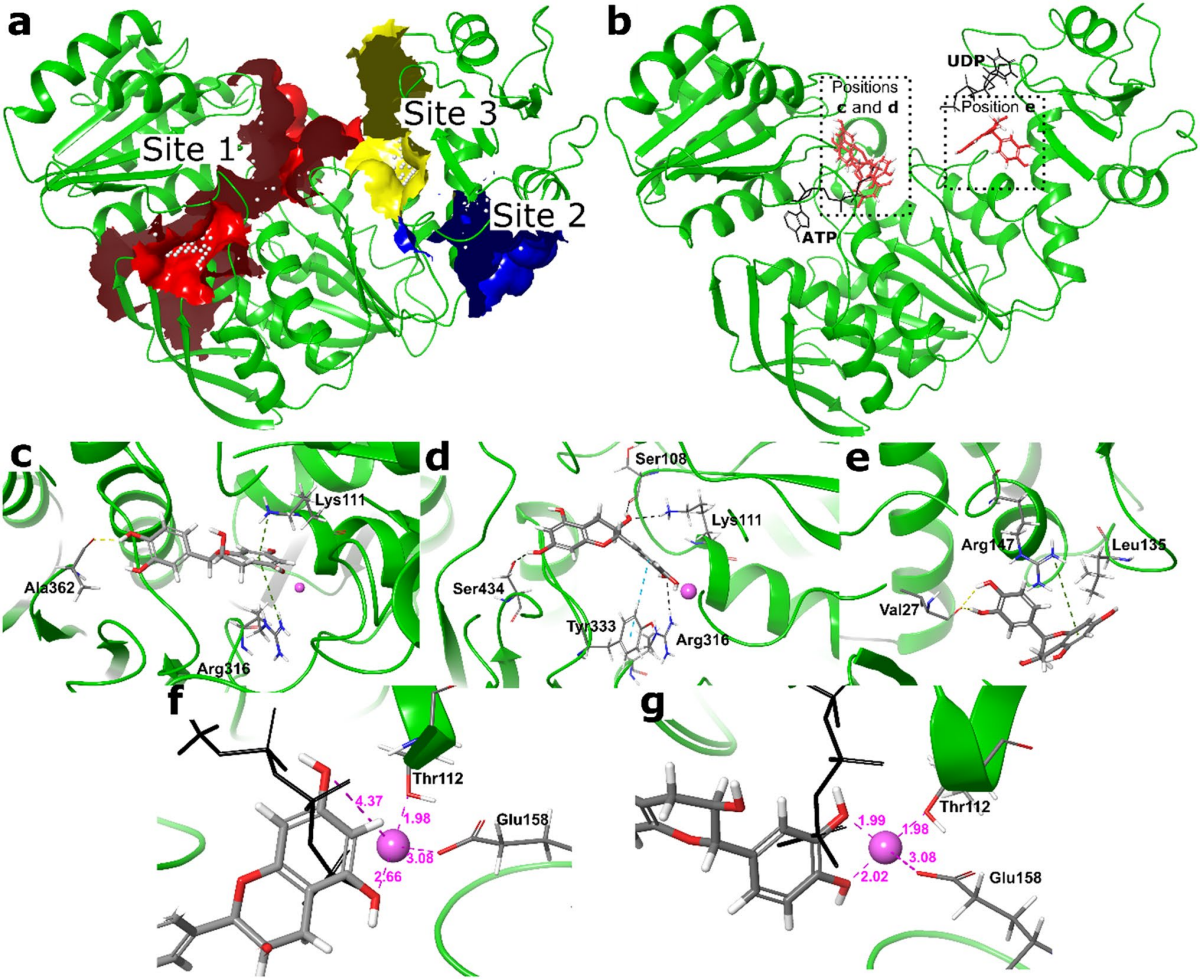
Molecular modelling results. **a** Ligandable binding sites identified with SiteMap; **b** three binding positions of (–)-epicatechin (**21**) bound to MurF. Native ATP- and UDP-binding conformations are presented in black sticks for comparison; **c**, **d** and **e** 3D binding modes of (–)-epicatechin (**21**); **f** and **g** magnified view highlighting interactions between Mg and the phenolic groups of (–)-epicatechin (**21**). The purple numbers indicate the distances in angstroms (Å) between Mg, Thr112, Glu158, and the two phenolic groups of ring B. ATP is shown as a black line presentation for reference. Interactions with surrounding amino acids are visualized as coloured dotted lines. Interactions with surrounding amino acids are shown as coloured dotted lines. Created with Pymol 2.0 and Inkscape 1.0.1

MurF binding sites were identified and evaluated using SiteMap. SiteMap calculates SiteScore and DScore for each binding site and determines their basic physicochemical properties. A total of seven binding sites were identified and are listed in Table 3. However, only three binding sites were found to be ligandable and druggable with SiteScore and DScore values each greater than 0.8, a treshold suggested and validated by the software developers (Halgren, 2009). The three sites are shown in Fig. 2a. Site 1 is the largest binding pocket and binds both ATP, the d-Ala-d-Ala dipeptide and tripeptide portions of the UM3DAP substrate. In contrast, sites 2 and 3 are much smaller. Site 3 binds the UDP part of the UM3DAP substrate and site 2 is not involved in substrate binding.

**Table 3:** Analysis of the druggability and ligandability of the binding sites with sitemap

Title	SiteScore	Dscore	Volume (Å^3^)
Site 1	1.044	0.936	872
Site 2	0.897	0.888	139
Site 3	0.874	0.881	236
Site 4	0.677	0.633	92
Site 5	0.656	0.63	72
Site 6	0.655	0.657	105
Site 7	0.570	0.301	52

### Docking analysis

To structurally rationalize the binding of (–)-epicatechin (**21**) to the enzyme MurF, computational analysis was performed using the docking software Glide from Schrodinger. The compounds were prepared using LigPrep from the Schrödinger Suite and docked to the homology model of MurF from *E. coli*. Two grid boxes were used for docking—one including site 1 and one including sites 2 and 3 (see SiteMap analysis).

The 3D representation of the highest scoring binding modes of (–)-epicatechin (**21**) can be found in Fig. 2b-g, where the protein structure is shown as a green ribbon diagram. For comparison, ATP from the native structure and UDP from a different crystal structure of MurF (PDB accession code 4QDI) are also shown as black stick representations. Figure 2b shows that **21** was able to bind to two different regions of the enzyme in our model, site 1 and site 3, previously identified by Sitemap. We identified two almost equally favourable binding orientations of **21** (labeled c and d) in site 1, and neither of them overlapped with the UM3DAP or the d-Ala-d-Ala region of the enzyme. Both binding orientations were located at the entrance of the cleft where ATP binds and partially overlapped with its γ-phosphate group (Fig. 2f,g). The amino acids surrounding the ligand and forming non-covalent interactions with **21** are shown as stick presentations in Fig. 2c–e. In the first binding orientation (position c), hydrogen bonds were formed between the phenolic hydroxyl group and Ala362 and π-cation interactions with Lys111 and Arg316. In the second binding orientation (position d), hydrogen bonds were formed between the phenolic hydroxyl groups and residues Ser434 and Arg316, and between the alcoholic hydroxyl group and residues Ser108 and Lys111. In addition, a π–π T-shaped interaction occurred between the phenyl ring of Tyr333 and the B ring of **21.** In both orientations, a salt bridge was formed between the phenolic hydroxyl group and the magnesium cofactor. It is noteworthy that the docking score for position d was only slightly higher than for position c, while there was a clear disparity in the interactions with Mg. Position d had more favourable interactions in closer proximity to Mg. In position d, Mg was in a complex between the two catecholic hydroxyl groups of ring B at an approximate distance of 2 Å, as shown in Fig. 2g. In position c, however Mg was positioned slightly below the plane of ring B, further away from the two 1,3-hydroxyl groups of ring B, resulting in distances of 2.66 and 4.37 Å, as shown in Fig. 2f, which made this interaction less favourable. For binding at site 3 (position e), only one orientation was found in which **21** formed two hydrogen bonds between Val27 and the phenolic groups of **21** as well as π-cation interaction with Arg147. Docking analysis of (–)-quercitrin (**10**) is shown in the SI.

## Discussion

Flavonoids are a large and structurally diverse class of polyphenolic compounds containing a heterocyclic ring. They are found in relatively large amounts in a variety of foods. The antimicrobial properties of some dietary preparations containing flavonoids have been documented in the world’s oldest medical literature since antiquity. Not surprisingly, in recent years, many flavonoids have been characterized for their antibacterial activities against a wide range of common Gram-positive and Gram-negative pathogens in vitro [7, 30].

In this study, biochemical screening of 24 flavonoids and related compounds including a silymarin extract against Mur ligases C, D, E, and F was performed. This led to the discovery of three MurF inhibitors, quercitrin (**10**), myricetin (**12**), and (–)-epicatechin (**21**). Most flavonoids did not inhibit any of the other enzymes, and for some compounds, the inhibition results were not reliable enough to detect or confirm inhibition at a concentration of 100 µM.

This degree of observed specificity is surprising, as flavonoids are known to inhibit a variety of enzymes, including ATP-dependent enzymes. Even the three compounds shown to be active in our assay are known to inhibit several other enzymes [31]. For example, the most active compound in our assay, (–)-epicatechin (**21**), has been reported to inhibit the activity of RNAse A [32], ribonuclease A [33], HIV-1 reverse transcriptase [34] and cyclooxygenase [35]. A significant degree of selectivity has been reported only for a limited number of different enzyme pairs to date [36–38].

Because MurC, D, E, and F have similar quaternary structures, particularly in the ATP-binding region, it seemed unlikely that these flavonoids could act as ATP-competitive inhibitors. Therefore, the binding kinetics of the most potent flavonoid (–)-epicatechin (**21**) were further investigated. It was found that (–)-epicatechin (**21**) acts as a mixed inhibitor with respect to coenzyme ATP and an uncompetitive inhibitor with both substrates, UM3DAP and d-Ala-d-Ala dipeptide. Both types of inhibition were partial, as the enzyme-substrates-inhibitor complex remained catalytically active after the inhibitor bound to the enzyme. These two types of inhibition suggest that (–)-epicatechin (**21**) binds to the enzyme form in which both substrates are already bound, at a site different from the ATP-binding site. This allosteric binding site could theoretically be close or further away to the ATP binding site.

We next analyzed the structural features necessary for activity. We evaluated several flavonoids that differed in their main skeleton, shape, number and position of hydroxyl groups, mode of alkylation and glycosylation. Although previous studies have emphasized the importance of the geometry and nature of the flavonoid skeleton for ATP-dependent enzymes [39, 40], we did not find a significant effect of the flatness or size of the flavonoid skeleton on activity. The three flavonoids that inhibited MurF were structurally distinct and belonged to different structural groups. The substitution pattern and the nature of the substituents appeared to have the greatest influence on activity, and the three active compounds shared some common structural patterns. For instance, they all have a substituent at position 3 of the C ring and had at least two unsubstituted hydroxyl groups on each of the A and B rings. Upon comparison of these features with those of the inactive compounds, we found that most of the inactive compounds lacked at least one of these two features, except for compounds **7**–**9**. The importance of the number and substitution pattern of the flavonoid hydroxyl groups for inhibitory properties has also been reported for other enzymes [33, 38]. However, these patterns were not sufficient to fully explain why some flavonoids, e.g., quercetin (**14**), leucocyanidin (**20**) and (+)-catechin (**22**), were inactive.

To further rationalize the structural features of the most active compounds and to determine their possible binding site, a docking analysis of (–)-epicatechin (**21**) was performed. However, because the binding kinetics indicated that this compound binds to MurF in a closed conformation, the apo form of the enzyme, which is currently the only available form of MurF from *E. coli*, could not be used. Therefore, a homology model was constructed and to avoid docking to unligandable binding sites, binding pocket detection was performed using SiteMap software. Of the seven identified binding sites, only three were found to be ligandable enough to be used for the further docking procedure. Two possible binding sites were found, one near the ATP-binding site and the other close to the UM3DAP binding site. Since the Hill coefficient was calculated to be 1.0, indicating no substrate binding cooperativity, it can be assumed that two molecules of **21** independently bind to the enzyme without affecting each other. In addition, three possible binding conformations were identified, and a close examination of the hydrogen bonds revealed that the hydroxyl groups in the 5- and 7- positions of the A-ring and in the 3′- and 4′- positions of the B-ring are important for binding to the enzyme and interact with the surrounding amino acids or a magnesium cofactor. In addition, π-cation interactions between the A-ring and surrounding positively charged residues were found to be important factors in the binding efficiency of these flavonoids to MurF. The idea that flavonoids interact with a variety of different orientations and different binding sites is not unusual and has been well documented for several protein kinases [41, 42], Pim-1 [43], CDK1 [44] and DNA gyrase [45]. This is likely due to the large number of functional groups flavonoids have that can form hydrogen bonds with the residues of each enzyme. However, it should be noted that the different binding modes of **21** in MurF are only a hypothesis that needs to be confirmed by crystallographic analysis.

We then investigated whether any of these compounds inhibited the growth of *E. coli* or *S. aureus*. Several compounds were found to inhibit the growth of *S. aureus* (compounds **2**, **3**, **14**, **16**, and **23**) with **23** showing the highest inhibitory activity with a MIC of 31 µM. None of these compounds were active in the enzyme assay, suggesting that their antibacterial activity was not due to inhibition of Mur ligases C, D, E, or F, but rather a different target or targets. However, quercitrin (**10**), myricetin (**12**), and (–)-epicatechin (**21**), which inhibited MurF, were found to be inactive against both bacterial strains. This discrepancy between the activity on the isolated enzyme and the bacteria is not unusual and could be explained by intrinsic bacterial resistance mechanisms, such as active antimicrobial efflux, decreased penetration of the drug into cells, and enzymatic metabolism of the antimicrobial agents [46]. For catechins, it has already been reported that the strong negative charge on the outer membrane of Gram-negative bacteria repels the negative phenolate ions [47] and favours primary amines and amphiphilic molecules [48]. In addition, there is a discrepancy between our results and literature data reporting that myricetin and quercitrin inhibit the growth of *S*. *aureus* [49, 50]. However, these reports were performed on different bacterial strains and used different assay methods, which could account for the discrepancies in the results.

The results of our study provide a foundation for the development of novel lead compounds possessing antibacterial activity. While the activity of these compounds against isolated enzymes was found to be weak and no antibacterial activity was detected, they could potentially be used as templates for the development of antibacterial lead compounds. By modifying or synthesizing structurally similar analogs, it may be possible to create compounds with increased potency, as has been demonstrated with (+)-catechin [51] and myricetin [52] derivatives that possess antibacterial activity. Additionally, knowledge about the structural requirements necessary for activity could be used to construct a pharmacophore model that would enable the design of structurally diverse compounds with superior pharmacological properties.

## Conclusion

In this manuscript, we present the screening results of 25 flavonoids and related compounds, including a silymarin extract, against four bacterial enzymes (MurC, D, E and F) involved in the synthesis of PG. Biochemical assays confirmed the inhibitory activity of three compounds—quercitrin (**10**), myricetin (**12**), and (–)-epicatechin (**21**)—against MurF with IC_50_ values of 143µM, 139µM, and 92µM, respectively. Compound **21** was found to be a mixed inhibitor with respect to coenzyme ATP and an uncompetitive inhibitor with both substrates, UM3DAP and d-Ala-d-Ala dipeptide. To investigate the binding of these flavonoids, *in-silico* analysis of the binding sites in MurF was conducted along with docking analysis of the most potent inhibitor, **21**. Two possible binding sites for **21** were identified, one close to the ATP-binding site and the other adjacent to the UM3DAP binding site. A detailed study of the interactions between (–)-epicatechin and the surrounding amino acids highlighted the importance of the aromatic A ring and the hydroxyl groups at positions 5, 7, and 3′ and 4′ of the A and B rings, respectively. Our research findings provide a starting point for the discovery of optimized MurF inhibitors with potential antibacterial activity.

### Supplementary Information

Below is the link to the electronic supplementary material.

Supplementary material 1 (DOCX 3833.6 kb)
