# Supplementary material for: Mur ligase F as a new target for the flavonoids quercitrin, myricetin, and (–)-epicatechin
Source: J Comput Aided Mol Des. 2023 Oct 5;37(12):721–33. doi: 10.1007/s10822-023-00535-z (PMC10618370; doi:10.1007/s10822-023-00535-z)
Supplement: Supplementary file 1 — Supplementary material 1 (DOCX 3833.6 kb) [file 10822_2023_535_MOESM1_ESM.docx]

**SUPPORTING INFORMATION**

**for**

Mur Ligase F as a New Target for the Flavonoids Quercitrin, Myricetin and (-)-Epicatechin

Martina Hrast^1^ Irena Zdovc^2^ Nina Kočevar Glavač^3^ Stanislav Gobec^1*^, Rok Frlan^1*^

^1^ *University of Ljubljana, Faculty of Pharmacy, Department of Pharmaceutical Chemistry, Aškerčeva 7, 1000 Ljubljana, Slovenia,*

^2^ *University of Ljubljana, Veterinary Faculty, Institute of Microbiology and Parasitology, Gerbičeva ul. 60, Ljubljana, Slovenia,*

^3^ *University of Ljubljana, Faculty of Pharmacy, Ljubljana, Slovenia, Department of Pharmaceutical Biology, Aškerčeva 7, 1000 Ljubljana, Slovenia,*

* Corresponding authors: *University of Ljubljana, Faculty of Pharmacy, Aškerčeva 7, 1000 Ljubljana, Slovenia, Department of Pharmaceutical Chemistry, Ljubljana, Slovenia, rok.frlan@ffa.uni-lj.si,* *0000-0003-0956-5537;* [*stanislav.gobec@ffa.uni-lj.si*](mailto:stanislav.gobec@ffa.uni-lj.si)*, 0000-0002-9678-3083*

Contents

[1. SiteMap analysis 2](#_Toc143063887)

[2. Docking analysis of (-)-quercitrin (**10**) 3](#_Toc143063888)

[3. Compound characterization 3](#_Toc143063889)

[4. Lineweaver-Burk double-reciprocal plots 9](#_Toc143063890)

# SiteMap analysis

Table S1. Analysis of the druggability, ligandability and physicochemical properties of the binding sites with Sitemap.

| Title | SiteScore | Dscore | Volume (Å^3^) | exposure | enclosure | contact | phobic | philic | balance |
| --- | --- | --- | --- | --- | --- | --- | --- | --- | --- |
| Site 1 | 1.044 | 0.936 | 872 | 0.541 | 0.764 | 0.944 | 0.265 | 1.418 | 0.187 |
| Site 2 | 0.897 | 0.888 | 139 | 0.538 | 0.765 | 1.051 | 1.46 | 0.824 | 1.772 |
| Site 3 | 0.874 | 0.881 | 236 | 0.642 | 0.677 | 0.834 | 0.707 | 0.814 | 0.868 |
| Site 4 | 0.677 | 0.633 | 92 | 0.654 | 0.576 | 0.753 | 0.225 | 1.052 | 0.214 |
| Site 5 | 0.656 | 0.63 | 72 | 0.713 | 0.609 | 0.811 | 0.61 | 0.688 | 0.887 |
| Site 6 | 0.655 | 0.657 | 105 | 0.822 | 0.534 | 0.628 | 0.764 | 0.574 | 1.331 |
| Site 7 | 0.570 | 0.301 | 52 | 0.64 | 0.582 | 0.826 | 0.082 | 1.645 | 0.05 |

# Docking analysis of (-)-quercitrin (**10**)

The 3D representation of the best evaluated binding mode of (-)-quercitrin (**10**) can be seen in Figure 1 where the protein structure is shown as a green ribbon diagram. In our model, (-)-quercitrin (**10**) was able to bind with similar binding mode to (-)-epicatechin (**21**) at site 1, with the glycoside moiety pointing to the exterior of the enzyme and the phenolic ring positioned in close proximity of the γ-phosphate group of ATP. It did not bind to any other binding sites probably because of its larger volume due to the additional glycoside moiety. Compound **10** formed several hydrogen bonds with the surrounding amino acids, for example Glu363, Ala362, Tyr333, Lys 111 and Arg316 as well as a π-π stacking interaction with Tyr333. A salt bridge between the phenolic hydroxyl group and Mg is also formed.


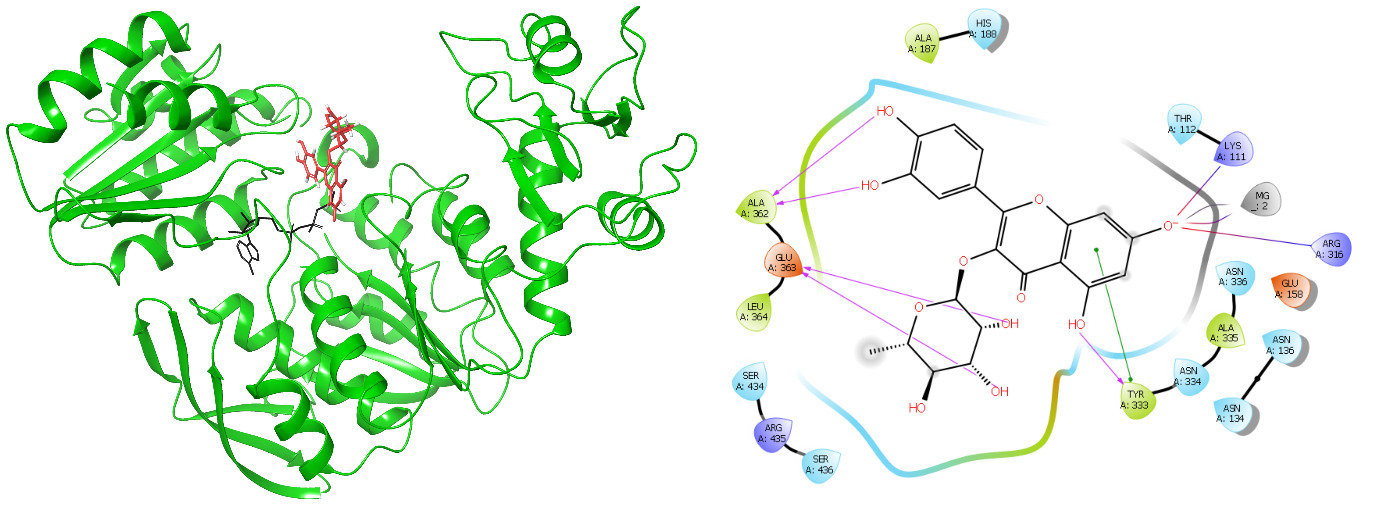


Figure S1. Docking of quercitrin (10) to MurF. (a) The 3D binding mode of (-)-quercitrin (10) at site 1. Native ATP binding is shown in black sticks for comparisson. (b) 2D interaction diagram between (-)-quercitrin (10) and surrounding residues shown as colored dotted lines.

# Compound characterization

Compounds were obtained from commercial sources (i.e., Acros Organics, Apollo Scientific, BLD Pharmatech, Fluorochem, MedChemExpress, Merck, TargetMol, TCI Europe) and used as received. ^1^H and ^13^C NMR spectra were recorded in a deuterated solvent on a Bruker Avance III 400 MHz spectrometer, operating at 400 and 101 MHz frequency. NMR spectra were measured at room temperature (25 °C) in deuterated DMSO. Chemical shifts (δ) are expressed in *parts per million* (*ppm*) referenced to TMS or residual solvent signals. Spectral data are reported in the following format: chemical shift (multiplicity, coupling constants, number of hydrogens). All coupling constants (*J*) are reported in Hertz. Compound purity was determined by HPLC analysis on Thermo Scientific Dionex UltiMate 3000 modular system (Thermo Fisher Scientific Inc.) with Waters Acquity UPLC^®^ HSS C18 SB column (2.1 × 50 mm, 1.8 µm) thermostated at 40 °C, injection volume, 1 µL; flow rate, 0.3 mL/min; detector λ, 254 nm; mobile phase A: 0.1% TFA (v/v) in water; mobile phase B: MeCN. Method: 0–5 min, 5%–30% B; 5-9 min, 100% B; 9-10 min, 100% B. Compounds are >95% pure by HPLC analysis, unless stated otherwise

**Myricetin** (**12**)

^1^H NMR (400 MHz, DMSO-d_6_): δ 12.51 (s, 1H), 10.80 (s, 1H), 9.37 (s, 1H), 9.24 (s, 2H), 8.83 (s, 1H), 7.25 (s, 2H), 6.38 (d, *J* = 2.0 Hz, 1H), 6.19 (d, *J* = 2.0 Hz, 1H) ppm. ^13^C NMR (101 MHz, DMSO-d_6_): δ 175.8, 163.9, 160.7, 156.1, 146.8, 145.7, 135.9, 135.9, 120.8, 107.2, 103.0, 98.2, 93.2 ppm. HPLC purity: 99.6 %.


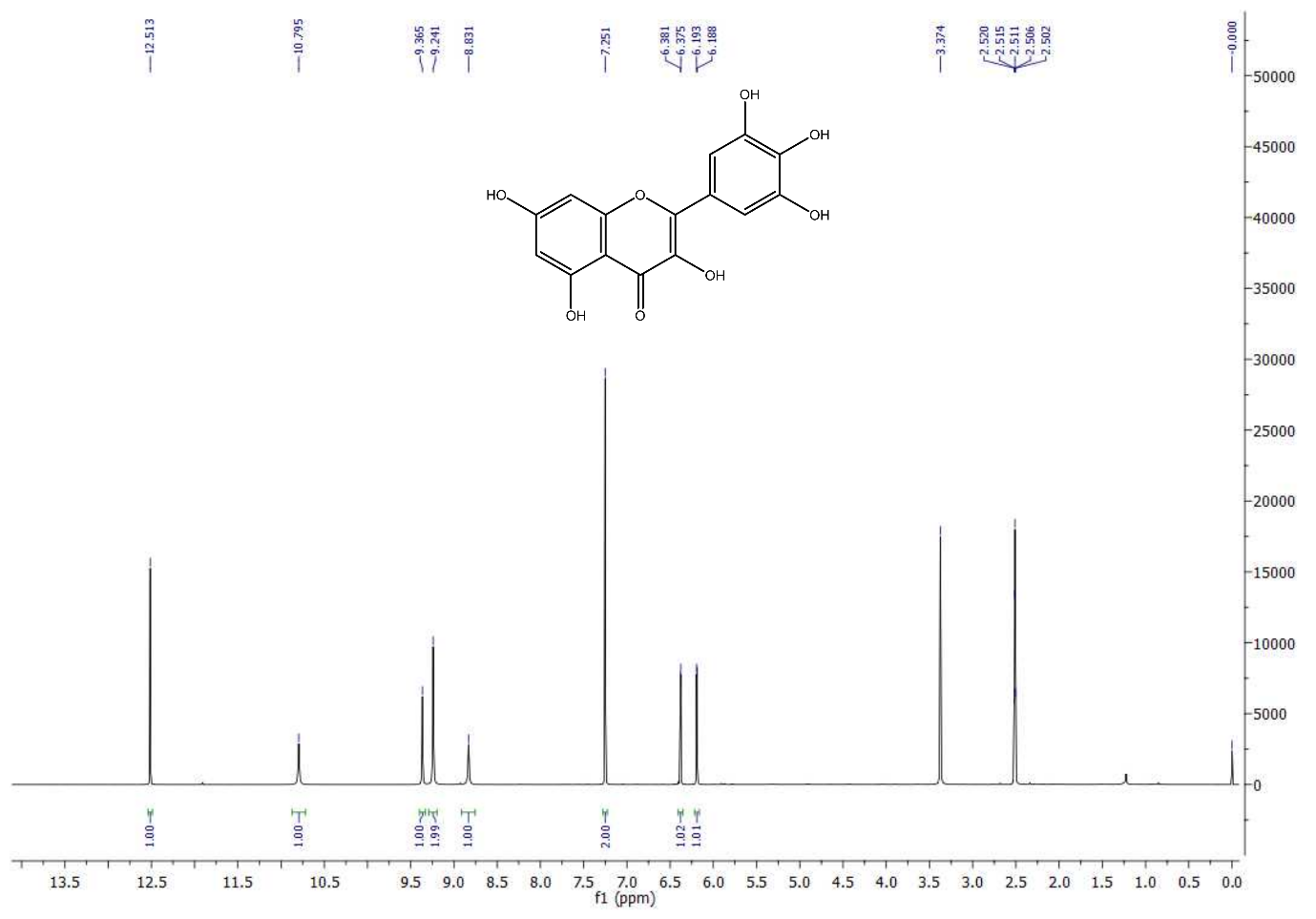


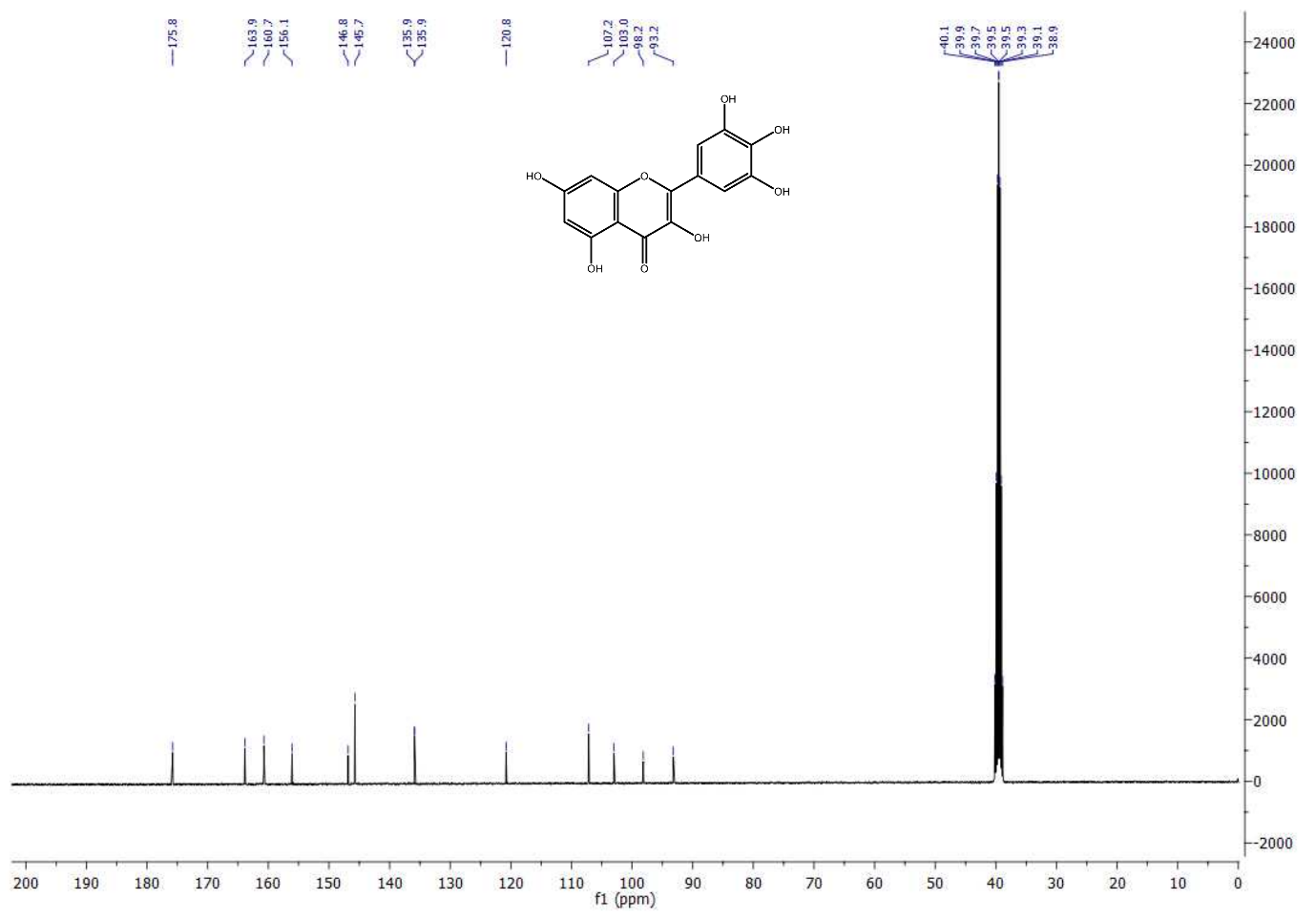


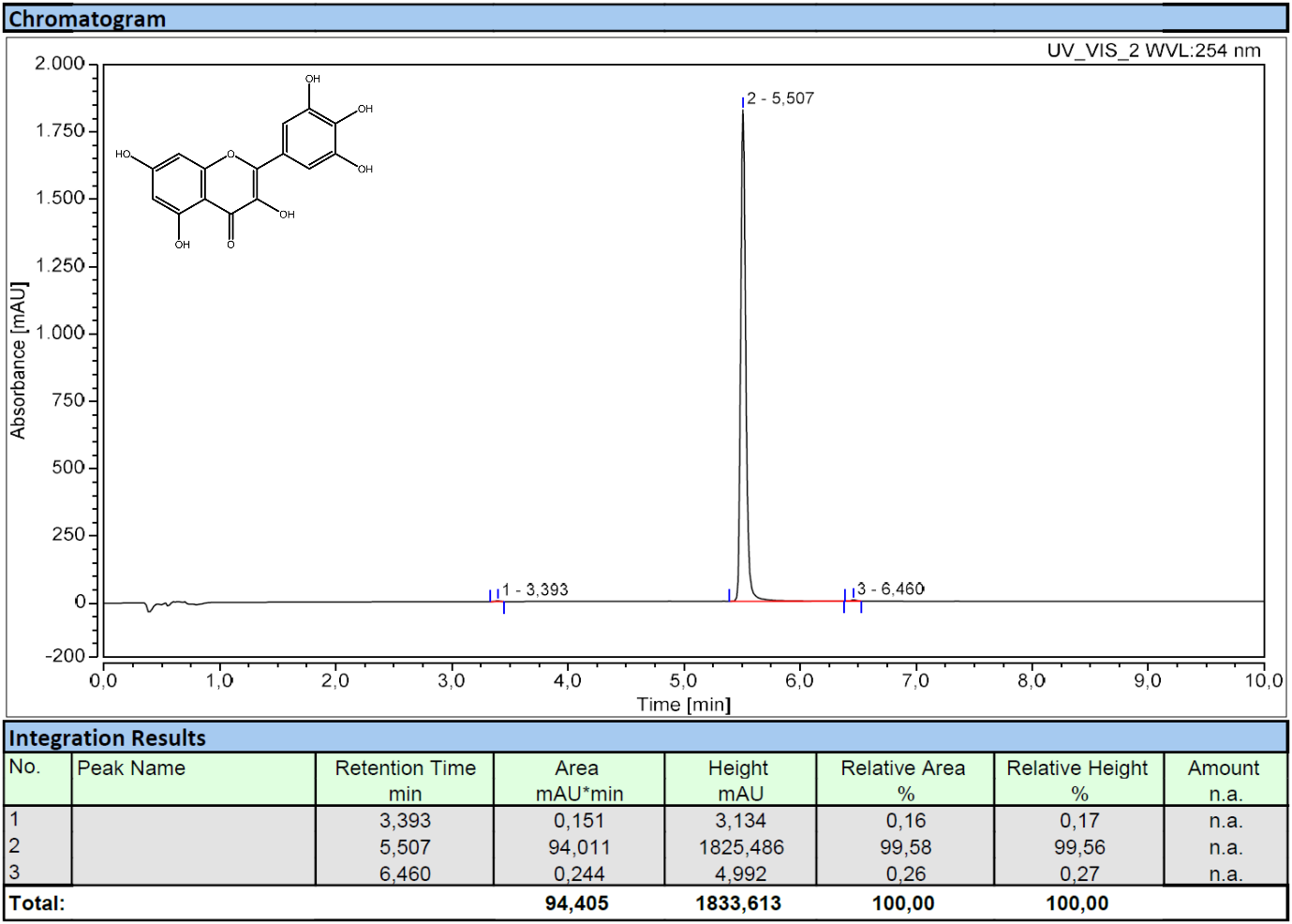


**Quercitrin** (**19**)

^1^H NMR (400 MHz, DMSO-d_6_): δ 12.67 (s, 1H), 10.89 (s, 1H), 9.73 (s, 1H), 9.36 (s, 1H), 7.30 (d, J = 2.1 Hz, 1H), 7.26 (dd, *J* = 8.3, 2.1 Hz, 1H), 6.87 (d, *J* = 8.3 Hz, 1H), 6.40 (d, *J* = 2.1 Hz, 1H), 6.21 (d, *J* = 2.1 Hz, 1H), 5.26 (d, *J* = 1.3 Hz, 1H), 4.96 (d, *J* = 4.3 Hz, 1H), 4.74 (d, *J* = 4.6 Hz, 1H), 4.63 (d, *J* = 5.8 Hz, 1H), 3.98 (t, *J* = 4.3 Hz, 1H), 3.51 (ddd, *J* = 8.9, 5.6, 3.4 Hz, 1H), 3.27 – 3.07 (m, 2H), 0.82 (d, *J* = 6.0 Hz, 3H) ppm. ^13^C NMR (101 MHz, DMSO-d_6_): δ 177.8, 164.2, 161.3, 157.3, 156.5, 148.4, 145.2, 134.2, 121.1, 120.7, 115.6, 115.5, 104.1, 101.8, 98.7, 93.6, 71.2, 70.6, 70.3, 70.1, 17.5 ppm. HPLC purity: 99.7 %.


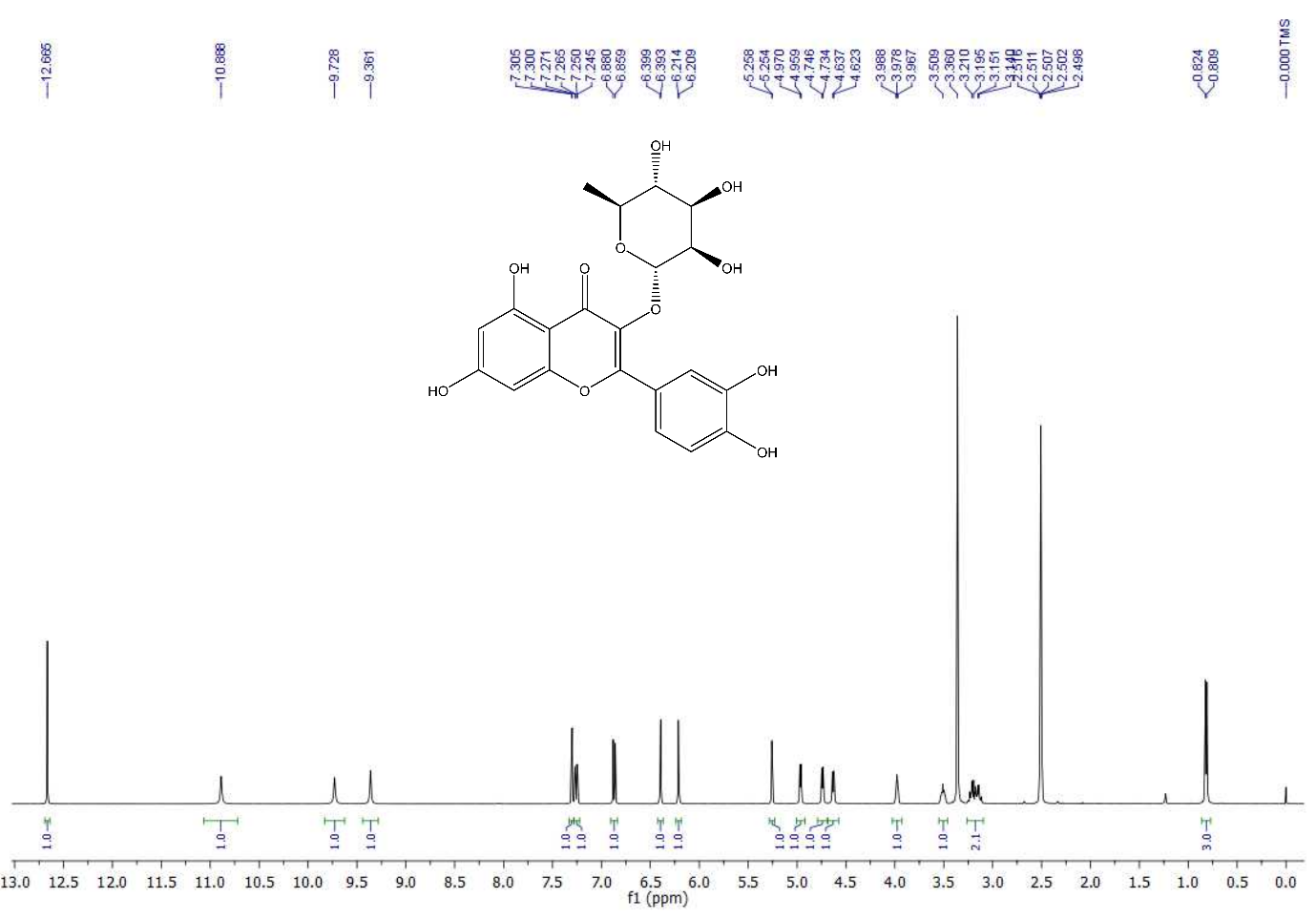


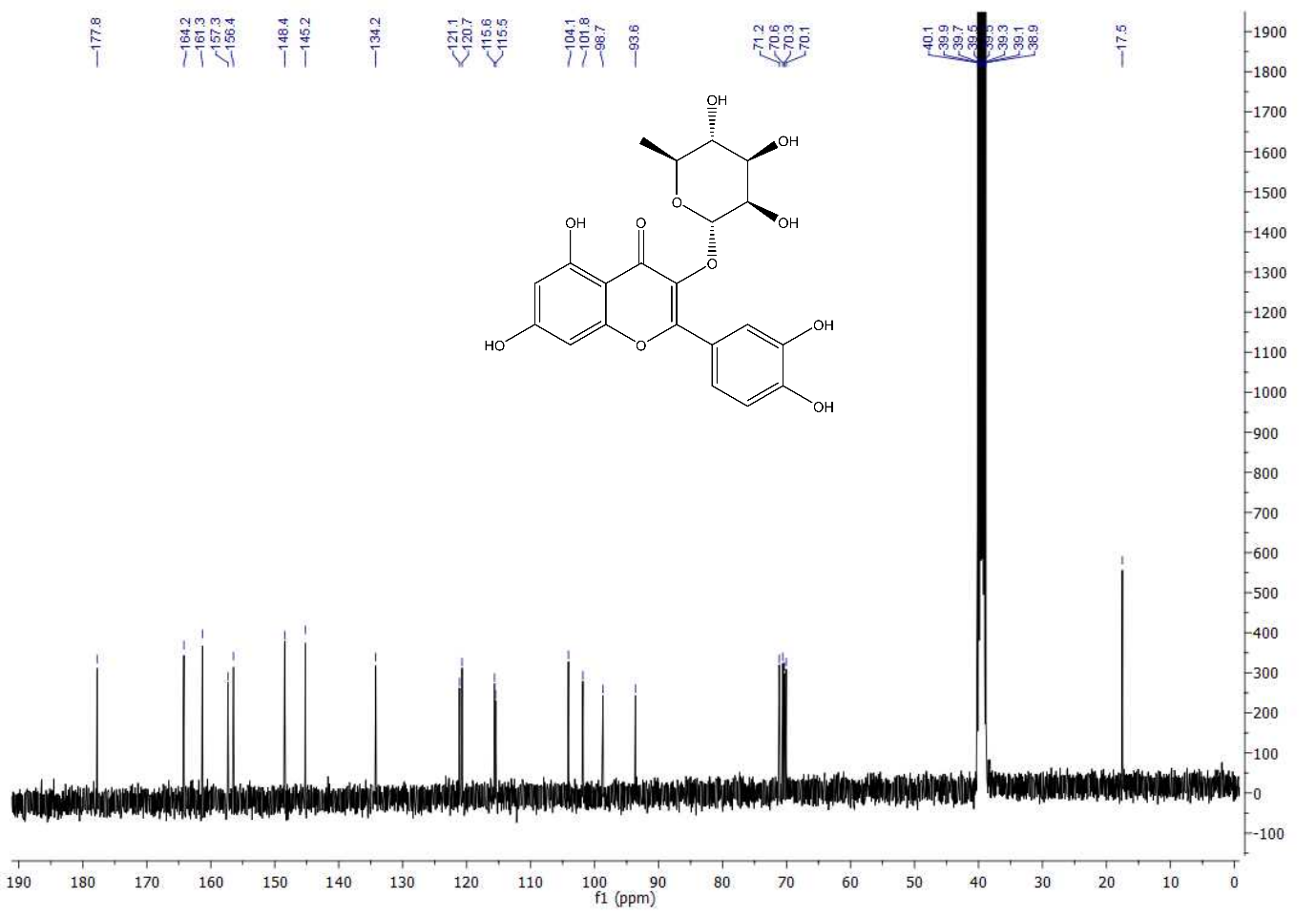


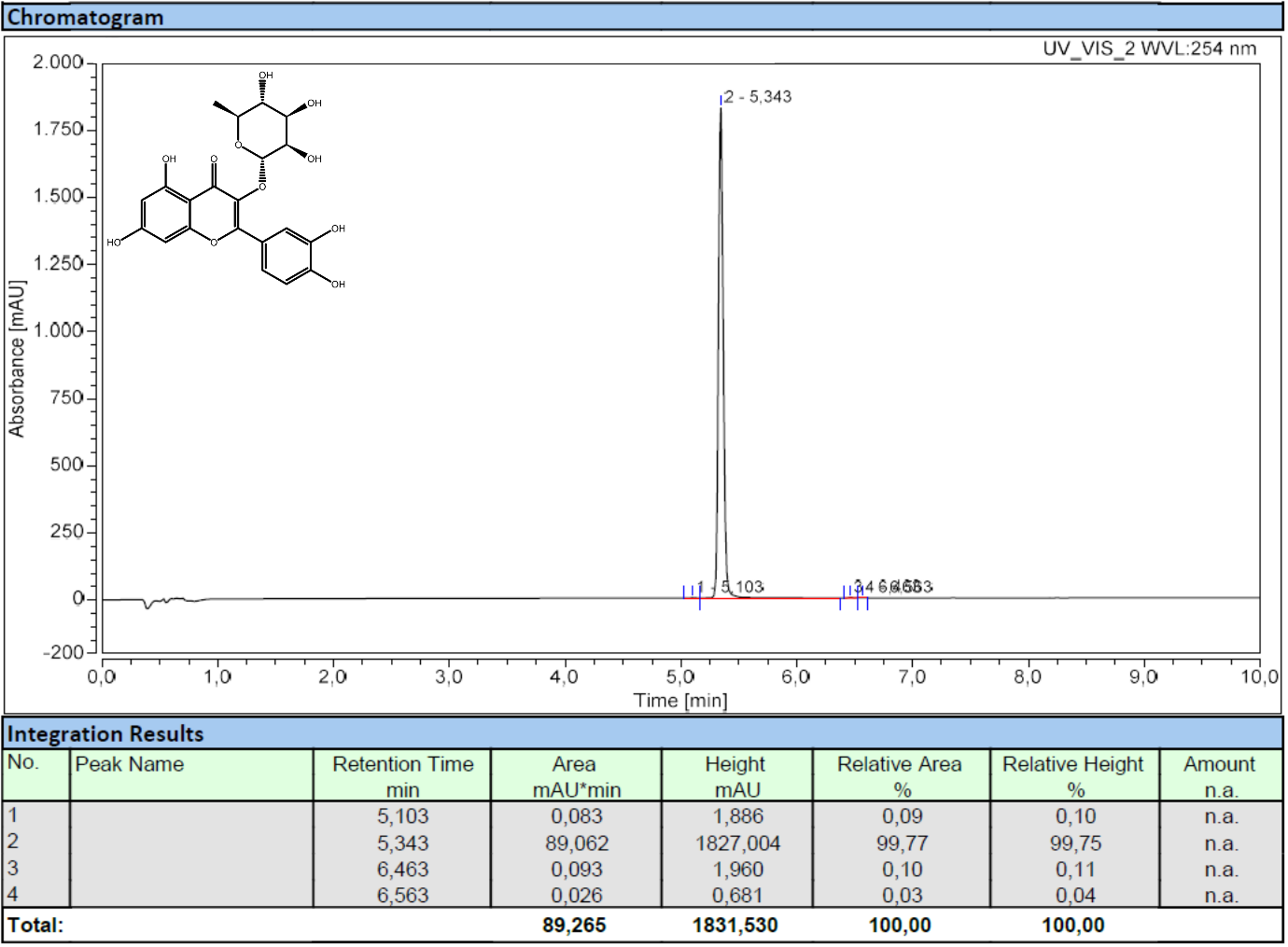


**(-)-epicatechin** (**21**)

^1^H NMR (400 MHz, DMSO-d_6_): δ 9.12 (s, 2H), 8.91 (s, 2H), 8.81 (s, 2H), 8.73 (s, 2H), 6.88 (d, *J* = 1.5 Hz, 2H), 6.69 – 6.60 (m, 4H), 5.88 (d, *J* = 2.3 Hz, 2H), 5.71 (d, *J* = 2.3 Hz, 2H), 4.73 (s, 2H), 4.67 (d, *J* = 4.6 Hz, 2H), 4.05-3.95(m, 2H), 2.67 (dd, J = 16.3, 4.4 Hz, 2H), 2.53 – 2.41 (m, 1H) ppm. ^13^C NMR (101 MHz, DMSO-d_6_): δ 156.5, 156.2, 155.8, 144.5, 144.4, 130.6, 118.0, 114.9, 114.8, 98.5, 95.0, 94.1, 78.1, 64.9, 28.2 ppm. HPLC purity: 96.8 %.


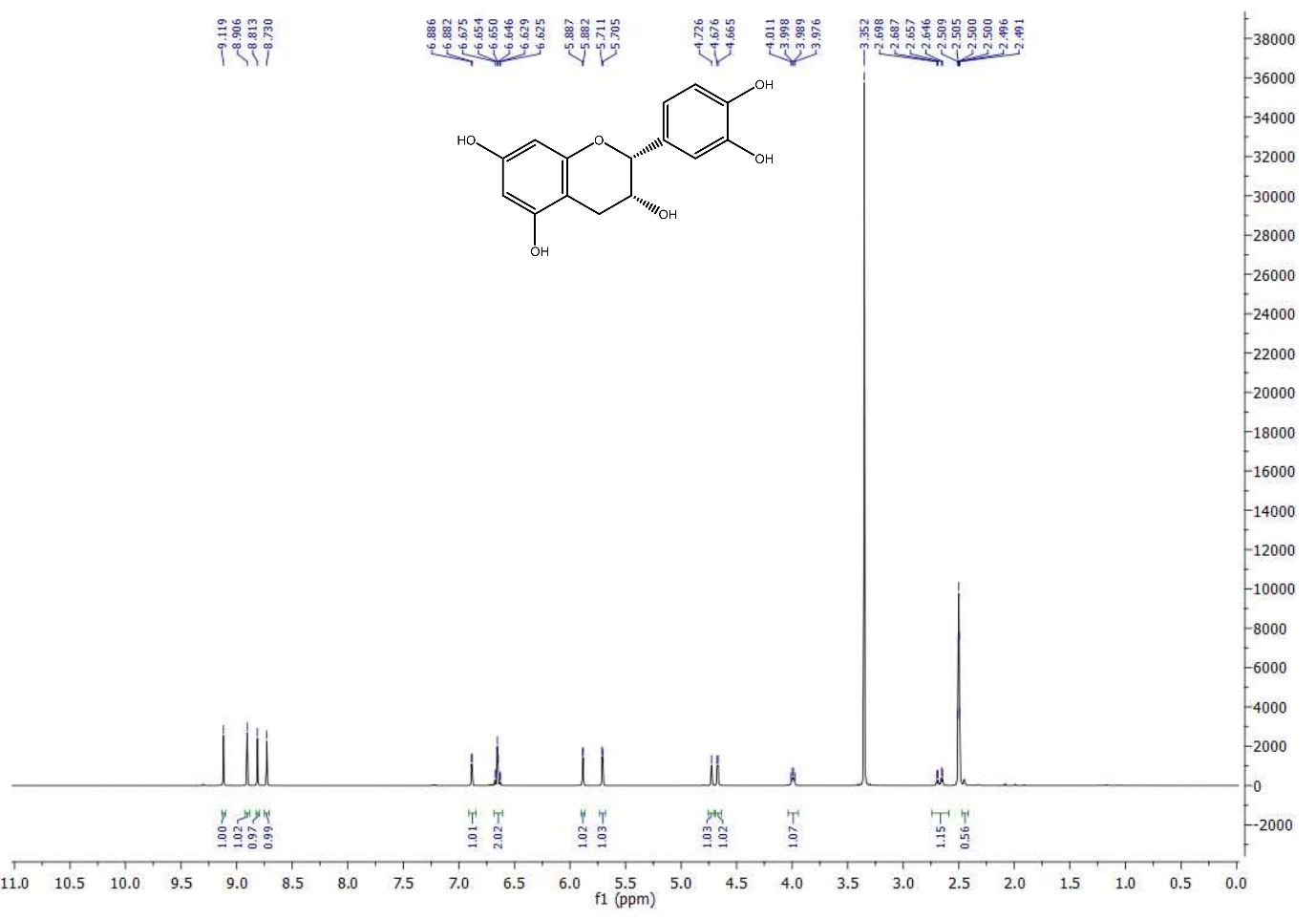


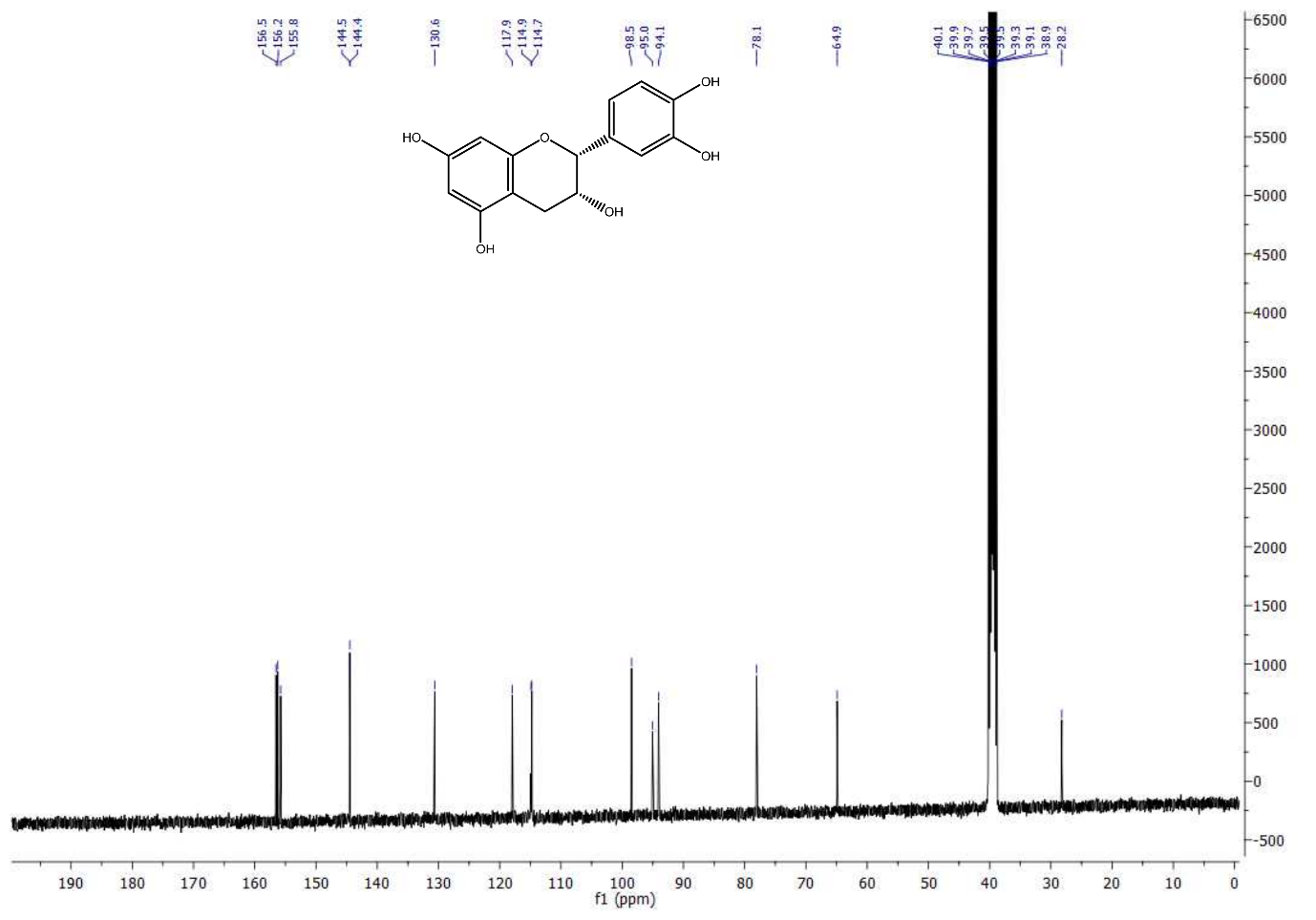

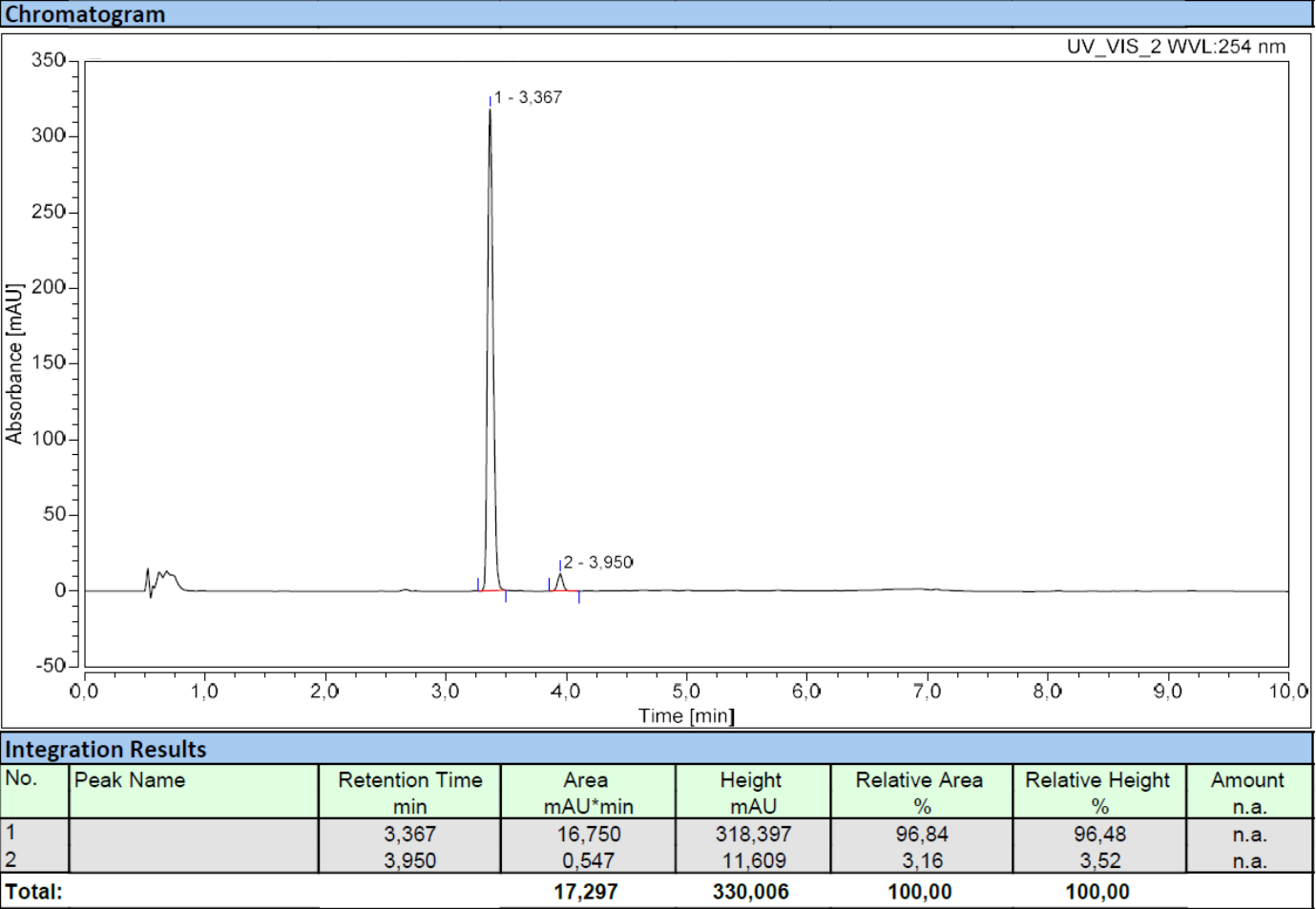


# Lineweaver-Burk double-reciprocal plots

**Figure S2:** Lineweaver-Burk plots of initial velocity of the MurF reaction as a function of the A) ATP; B) D-Ala-D-Ala, and C) UM3DAP.
